# (2-Amino­pyrimidine-κ*N* ^1^)diaqua(pyridine-2,6-dicarboxyl­ato-κ^3^ *O* ^2^,*N*,*O* ^6^)­nickel(II) monohydrate

**DOI:** 10.1107/S1600536810016843

**Published:** 2010-05-15

**Authors:** Masoumeh Tabatabaee

**Affiliations:** a Department of Chemistry, Islamic Azad University, Yazd Branch, Yazd, Iran

## Abstract

The reaction of Ni(NO_3_)_2_·6H_2_O with pyridine-2,6-dicarboxylic acid, NaOH and 2-amino­pyrimidine in aqueous solution leads to the formation of the title complex, [Ni(C_7_H_3_NO_4_)(C_4_H_5_N_3_)(H_2_O)_2_]·H_2_O. The Ni^II^ ion is coordinated by one N and two O atoms of the tridentate chelating pyridine-2,6-dicarboxyl­ate anion, one heterocyclic N atom of the 2-amino­pyrimidine ligand, and two water mol­ecules. The resulting geometry for the [NiN_2_O_4_] coordination environment can be described as distorted octa­hedral. One uncoord­inated water mol­ecule completes the asymmetric unit. Extensive O—H⋯O and N—H⋯O hydrogen-bonding inter­actions between the NH_2_ group of 2-amino­pyrimidine, carboxyl­ate groups, and coordinated and uncoordinated water mol­ecules contribute to the formation of a three-dimensional supra­molecular structure.

## Related literature

For transition metal complexes with 2-amino­pyrimidine, see: Ponticelli *et al.* (1999 ▶); Prince *et al.* (2003 ▶); Lee *et al.* (2003 ▶); Masaki *et al.* (2002 ▶). For related structures, see: Tabatabaee *et al.* (2008 ▶); Tabatabaee, Aghabozorg *et al.* (2009 ▶); Tabatabaee, Masoodpour *et al.* (2009 ▶); Tabatabaee, Sharif *et al.* (2009 ▶); Altin *et al.* (2004 ▶); Aghabozorg *et al.* (2007 ▶, 2008 ▶); Li *et al.* (2007 ▶).
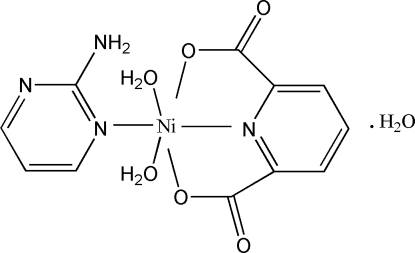


## Experimental

### 

#### Crystal data

[Ni(C_7_H_3_NO_4_)(C_4_H_5_N_3_)(H_2_O)_2_]·H_2_O
*M* *_r_* = 372.97
Monoclinic, 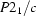

*a* = 9.6073 (8) Å
*b* = 10.2038 (10) Å
*c* = 14.6095 (15) Å
β = 102.677 (2)°
*V* = 1397.3 (2) Å^3^
*Z* = 4
Mo *K*α radiation
μ = 1.44 mm^−1^
*T* = 120 K
0.24 × 0.22 × 0.15 mm

#### Data collection

Bruker SMART 1000 CCD area-detector diffractometer
Absorption correction: multi-scan (*SADABS*; Bruker, 1998 ▶) *T*_min_ = 0.717, *T*_max_ = 0.810
12010 measured reflections
2725 independent reflections
2396 reflections with *I* > 2σ(*I*)
*R*_int_ = 0.029

#### Refinement

*R*[*F* ^2^ > 2σ(*F* ^2^)] = 0.056
*wR*(*F* ^2^) = 0.163
*S* = 1.01
2725 reflections
208 parameters
H-atom parameters constrained
Δρ_max_ = 0.65 e Å^−3^
Δρ_min_ = −0.51 e Å^−3^

### 

Data collection: *SMART* (Bruker, 1998 ▶); cell refinement: *SAINT-Plus* (Bruker, 1998 ▶); data reduction: *SAINT-Plus*; program(s) used to solve structure: *SHELXTL* (Sheldrick, 2008 ▶); program(s) used to refine structure: *SHELXTL*; molecular graphics: *SHELXTL*; software used to prepare material for publication: *SHELXTL*.

## Supplementary Material

Crystal structure: contains datablocks I, global. DOI: 10.1107/S1600536810016843/bh2281sup1.cif

Structure factors: contains datablocks I. DOI: 10.1107/S1600536810016843/bh2281Isup2.hkl

Additional supplementary materials:  crystallographic information; 3D view; checkCIF report

## Figures and Tables

**Table 1:** Hydrogen-bond geometry (Å, °)

*D*—H⋯*A*	*D*—H	H⋯*A*	*D*⋯*A*	*D*—H⋯*A*
N4—H4*B*⋯O1	0.86	2.07	2.867 (5)	153
N4—H4*C*⋯O4^i^	0.86	2.12	2.973 (6)	173
O1*W*—H1⋯O4^ii^	0.85	2.29	2.906 (7)	130
O1*W*—H1⋯O3^ii^	0.85	2.37	3.152 (5)	152
O2*W*—H3⋯O2^iii^	0.85	1.93	2.777 (4)	178
O2*W*—H4⋯O3*W*^iv^	0.85	1.84	2.685 (5)	173
O3*W*—H5⋯O2^v^	0.85	1.89	2.736 (4)	177
O3*W*—H6⋯O4^ii^	0.85	2.15	2.964 (6)	159
O3*W*—H6⋯O3^ii^	0.85	2.56	3.253 (5)	140

Symmetry codes: (i) 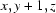; (ii) 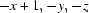; (iii) 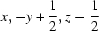; (iv) 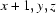; (v) 
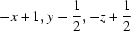
.

**Fig. 1.**
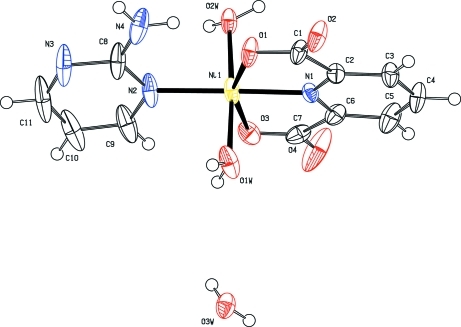
ORTEP-like view (30% probability level) of the title compound. The angle between the least-squares planes (N1/C2/C3/C4/C5/C6) and (N2/N3/N4/C8/C9/C10/C11) is 18.5 (1)°.

**Fig. 2.**
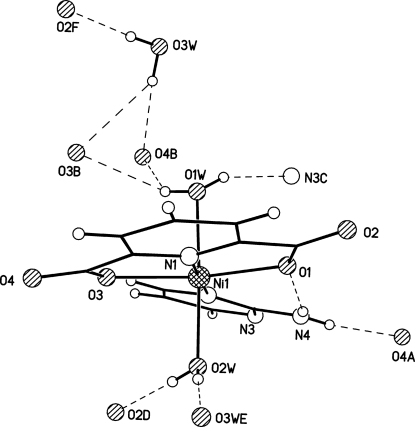
Fragment of hydrogen bonds (shown with dashed lines) in the title compound. Symmetry transformations used to generate equivalent atoms: #A x, y+1, z; #B -x+1, -y, -z; #C -x+1, -y+1, -z; #D x, -y+1/2, z-1/2; #E x+1, y, z; #F -x+1, y-1/2, -z+1/2.

**Fig. 3.**
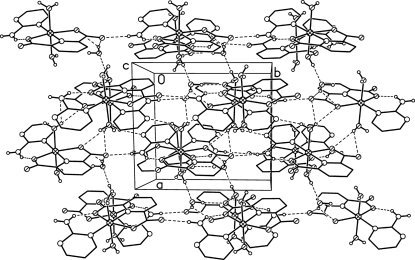
Fragment of crystal packing (view along crystallographic axes c). Only H atoms involved in hydrogen bonding are depicted. Hydrogen bonds are shown with dashed lines.

## supplementary crystallographic information

### Comment

Pyrimidine derivatives possess considerable biological activity and have been widely used in medicinal and industrial applications. The complexing ability of 2-aminopyrimidine derivatives with transition metal ions is of great interest. Several transition metal complexes of 2-aminopyrimidine with halide salts, M*X*_2_ (M= Pt, Pd, Cu, Mn, Co and Ni), have been synthesized and their crystal structures reported (Ponticelli *et al.*, 1999; Prince *et al.*, 2003; Lee *et al.*, 2003; Masaki *et al.*, 2002).

In continuation of our recent works on aminopyrimidine derivatives and hydrothermal synthesis of complexes (Tabatabaee *et al.*, 2008; Tabatabaee, Aghabozorg *et al.*, 2009; Tabatabaee, Masoodpour *et al*., 2009; Tabatabaee, Sharif *et al.*, 2009), in this communication we wish to report our results on the synthesis and characterization of the first complex of Ni^II^ with pyridine-2,6-dicarboxylic acid (pydcH_2_) and neutral 2-aminopyrimidine (amp), under hydrothermal conditions.

The title compound consists of [Ni(amp)(pydc)(H_2_O)_2_] and one uncoordinated water molecule (Fig. 1). The metal ion is hexacoordinated by nitrogen atom N1 and two oxygen atoms O1 and O3 of the pydc^2– ^fragment, which acts as a tridentate ligand, two oxygen atoms of two coordinated water molecules (O1W and O2W) and heterocyclic nitrogen atom of amp (N2). N1 and N2 atoms occupy the axial positions (shortest coordination bond lengths), while oxygen atoms form the equatorial plane. The crystal structure of a four-coordinated Cu^II^ complex with pyridine-2,6-dicarboxylate and 2-aminopyrimidine, formulated [Cu(amp)(pydc)].3H_2_O has been reported by Altin *et al.* (Altin *et al.*, 2004). Cu^2+^ ion in [Cu(amp)(pydc)] is four-coordinated with a pydc^2– ^tridentate ligand and N atom from 2-aminopyrimidine. In the title complex, N1—Ni1—N2 angle is deviated by 1.06° from linearity. The dihedral angle between the mean planes of the pyridine and pyrimidine rings is 18.5 (1)°, indicating that pyridine and pyrimidine ligands are almost parallel to each other. Ni—N distances of 1.975 (4) and 2.062 (4) Å and Ni—O distances [Ni1—O1W: 2.070 (4), Ni1—O2W: 2.076 (4), Ni1—O1: 2.117 (3) and Ni1—O7: 2.136 (4) Å] are consistent with the corresponding data reported in the literature (Aghabozorg *et al.*, 2007; Li *et al.*, 2007). According to bond lengths, bond angles and torsion angles, arrangement of the six donor atoms around Ni1 is distorted octahedral.

The extensive O—H···O, N—H···O hydrogen bonding interactions (Table 1) between complex and uncoordinated water molecule (Fig. 2) play an important role in stabilizing the crystal (Aghabozorg *et al.*, 2008; Tabatabaee, Aghabozorg *et al.*, 2009; Tabatabaee, Masoodpour *et al.*, 2009; Tabatabaee, Sharif *et al.*, 2009) and the formation of a three dimensional supramolecular crystal structure (Fig. 3).

### Experimental

Pyridine-2,6-dicarboxylic acid (0.167 g, 1 mmol) was dissolved in 10 ml of deionized water containing 0.08 g (2 mmol) of NaOH, and stirred for 30 min. at room temperature. A water solution of Ni(NO_3_)_2_.6H_2_O (0.29 g, 1 mmol) and 2-aminopyrimidine (0.095 g, 1 mmol) were added to the pyridine-2,6-dicarboxylic acid solution. Reaction mixture was placed in a Parr-Teflon lined stainless steel vessel. It was sealed and heated to 403 K for 8 h. Blue crystals of the complex were obtained upon slow cooling (Yield: 88%).

### Refinement

The H atoms bonded to O and N atoms were found in a difference map and normalized to distances of 0.86 and 0.85 Å, and positions of other H atoms were calculated. All hydrogen atoms were refined in isotropic approximation using a riding model, with *U*_iso_(H) parameters equal to 1.5 *U*_eq_(Oi), 1.2 *U*_eq_(Ni) and 1.2 *U*_eq_(Ci), where *U*(Xi) are the equivalent thermal parameters of the atoms to which the corresponding H atoms are bonded.

### Crystal data

**Table:**

[Ni(C_7_H_3_NO_4_)(C_4_H_5_N_3_)(H_2_O)_2_]·H_2_O	*F*(000) = 768
*M**_r_* = 372.97	*D*_x_ = 1.773 Mg m^−^^3^
Monoclinic, *P*2_1_/*c*	Mo *K*α radiation, λ = 0.71073 Å
Hall symbol: -P 2ybc	Cell parameters from 896 reflections
*a* = 9.6073 (8) Å	θ = 2.9–25.0°
*b* = 10.2038 (10) Å	µ = 1.44 mm^−^^1^
*c* = 14.6095 (15) Å	*T* = 120 K
β = 102.677 (2)°	Prism, blue
*V* = 1397.3 (2) Å^3^	0.24 × 0.22 × 0.15 mm
*Z* = 4	

### Data collection

**Table:**

Bruker SMART 1000 CCD area-detector diffractometer	2725 independent reflections
Radiation source: fine-focus sealed tube	2396 reflections with *I* > 2σ(*I*)
graphite	*R*_int_ = 0.029
φ and ω scans	θ_max_ = 26.0°, θ_min_ = 2.2°
Absorption correction: multi-scan (*SADABS*; Bruker, 1998)	*h* = −11→11
*T*_min_ = 0.717, *T*_max_ = 0.810	*k* = −12→12
12010 measured reflections	*l* = −18→17

### Refinement

**Table:**

Refinement on *F*^2^	Primary atom site location: structure-invariant direct methods
Least-squares matrix: full	Secondary atom site location: difference Fourier map
*R*[*F*^2^ > 2σ(*F*^2^)] = 0.056	Hydrogen site location: mixed
*wR*(*F*^2^) = 0.163	H-atom parameters constrained
*S* = 1.01	*w* = 1/[σ^2^(*F*_o_^2^) + (0.07*P*)^2^ + 9.*P*] where *P* = (*F*_o_^2^ + 2*F*_c_^2^)/3
2725 reflections	(Δ/σ)_max_ < 0.001
208 parameters	Δρ_max_ = 0.65 e Å^−^^3^
0 restraints	Δρ_min_ = −0.51 e Å^−^^3^
0 constraints	

### Fractional atomic coordinates and isotropic or equivalent isotropic displacement parameters (Å^2^)

**Table:**

	*x*	*y*	*z*	*U*_iso_*/*U*_eq_	
Ni1	0.71391 (7)	0.20931 (6)	0.07096 (4)	0.0268 (2)	
N1	0.7944 (4)	0.0931 (3)	0.1775 (2)	0.0212 (8)	
N2	0.6262 (5)	0.3303 (4)	−0.0398 (3)	0.0364 (11)	
N3	0.5712 (6)	0.5362 (6)	−0.1177 (3)	0.0574 (17)	
N4	0.7236 (6)	0.5244 (4)	0.0275 (3)	0.0451 (13)	
H4B	0.7678	0.4812	0.0758	0.054*	
H4C	0.7332	0.6080	0.0254	0.054*	
O1	0.7779 (4)	0.3446 (3)	0.1821 (2)	0.0328 (8)	
O2	0.8528 (5)	0.3529 (3)	0.3379 (2)	0.0395 (9)	
O3	0.6800 (4)	0.0198 (4)	0.0080 (2)	0.0328 (8)	
O4	0.7485 (7)	−0.1853 (4)	0.0367 (3)	0.0740 (18)	
C1	0.8233 (6)	0.2929 (4)	0.2627 (3)	0.0289 (11)	
C2	0.8404 (5)	0.1458 (4)	0.2621 (3)	0.0229 (9)	
C3	0.8944 (5)	0.0683 (5)	0.3388 (3)	0.0290 (10)	
H3A	0.9272	0.1048	0.3979	0.035*	
C4	0.8982 (6)	−0.0660 (5)	0.3249 (4)	0.0376 (12)	
H4A	0.9347	−0.1210	0.3753	0.045*	
C5	0.8481 (7)	−0.1188 (5)	0.2367 (4)	0.0394 (13)	
H5A	0.8490	−0.2088	0.2272	0.047*	
C6	0.7967 (5)	−0.0346 (4)	0.1630 (3)	0.0271 (10)	
C7	0.7374 (5)	−0.0716 (5)	0.0620 (3)	0.0307 (11)	
C8	0.6402 (7)	0.4615 (6)	−0.0441 (3)	0.0416 (15)	
C9	0.5438 (7)	0.2718 (7)	−0.1134 (4)	0.0497 (17)	
H9A	0.5343	0.1812	−0.1121	0.060*	
C10	0.4716 (7)	0.3385 (8)	−0.1914 (4)	0.060 (2)	
H10A	0.4143	0.2962	−0.2424	0.072*	
C11	0.4907 (8)	0.4728 (8)	−0.1885 (4)	0.068 (3)	
H11A	0.4436	0.5216	−0.2397	0.082*	
O1W	0.5159 (4)	0.2003 (4)	0.1048 (2)	0.0419 (10)	
H1	0.4722	0.1549	0.0585	0.063*	
H2	0.4721	0.2693	0.1148	0.063*	
O2W	0.9039 (4)	0.2190 (3)	0.0257 (2)	0.0266 (7)	
H3	0.8894	0.1987	−0.0320	0.040*	
H4	0.9753	0.1835	0.0619	0.040*	
O3W	0.1432 (4)	0.1177 (3)	0.1323 (2)	0.0320 (8)	
H5	0.1465	0.0351	0.1402	0.048*	
H6	0.1922	0.1248	0.0907	0.048*	

### Atomic displacement parameters (Å^2^)

**Table:**

	*U*^11^	*U*^22^	*U*^33^	*U*^12^	*U*^13^	*U*^23^
Ni1	0.0394 (4)	0.0241 (3)	0.0137 (3)	0.0101 (3)	−0.0012 (2)	−0.0018 (2)
N1	0.0236 (19)	0.0191 (18)	0.0180 (17)	0.0013 (14)	−0.0016 (14)	−0.0012 (14)
N2	0.057 (3)	0.038 (2)	0.0124 (18)	0.029 (2)	0.0047 (18)	−0.0002 (16)
N3	0.080 (4)	0.071 (4)	0.028 (3)	0.056 (3)	0.028 (3)	0.027 (3)
N4	0.087 (4)	0.024 (2)	0.029 (2)	0.020 (2)	0.023 (2)	0.0073 (18)
O1	0.062 (2)	0.0211 (16)	0.0139 (15)	0.0066 (16)	0.0060 (15)	0.0004 (13)
O2	0.081 (3)	0.0207 (17)	0.0148 (16)	−0.0061 (17)	0.0053 (16)	−0.0018 (13)
O3	0.0317 (18)	0.041 (2)	0.0225 (16)	0.0007 (15)	0.0000 (14)	−0.0072 (15)
O4	0.174 (6)	0.021 (2)	0.031 (2)	−0.021 (3)	0.029 (3)	−0.0078 (17)
C1	0.048 (3)	0.017 (2)	0.019 (2)	−0.004 (2)	0.001 (2)	0.0006 (17)
C2	0.028 (2)	0.019 (2)	0.020 (2)	−0.0006 (17)	0.0011 (17)	−0.0022 (17)
C3	0.040 (3)	0.027 (2)	0.017 (2)	0.003 (2)	0.0000 (19)	0.0004 (18)
C4	0.060 (4)	0.024 (2)	0.028 (3)	0.013 (2)	0.009 (2)	0.010 (2)
C5	0.071 (4)	0.020 (2)	0.031 (3)	0.003 (2)	0.018 (3)	0.000 (2)
C6	0.036 (3)	0.019 (2)	0.027 (2)	−0.0036 (19)	0.0095 (19)	−0.0023 (18)
C7	0.038 (3)	0.028 (2)	0.029 (2)	−0.013 (2)	0.014 (2)	−0.007 (2)
C8	0.067 (4)	0.043 (3)	0.020 (2)	0.038 (3)	0.020 (2)	0.012 (2)
C9	0.059 (4)	0.066 (4)	0.019 (2)	0.039 (3)	−0.002 (2)	−0.007 (2)
C10	0.052 (4)	0.111 (6)	0.017 (2)	0.056 (4)	0.005 (2)	−0.002 (3)
C11	0.082 (5)	0.107 (6)	0.021 (3)	0.077 (5)	0.023 (3)	0.025 (3)
O1W	0.041 (2)	0.067 (3)	0.0181 (16)	0.0300 (19)	0.0075 (15)	0.0021 (16)
O2W	0.043 (2)	0.0210 (16)	0.0131 (14)	0.0062 (14)	−0.0003 (13)	−0.0016 (12)
O3W	0.047 (2)	0.0229 (17)	0.0244 (16)	0.0005 (15)	0.0040 (15)	0.0003 (13)

### Geometric parameters (Å, °)

**Table:**

Ni1—N1	1.975 (4)	C2—C3	1.377 (6)
Ni1—N2	2.062 (4)	C3—C4	1.388 (7)
Ni1—O1W	2.070 (4)	C3—H3A	0.9300
Ni1—O2W	2.076 (4)	C4—C5	1.382 (7)
Ni1—O1	2.117 (3)	C4—H4A	0.9300
Ni1—O3	2.136 (4)	C5—C6	1.381 (7)
N1—C6	1.321 (6)	C5—H5A	0.9300
N1—C2	1.330 (5)	C6—C7	1.509 (6)
N2—C9	1.329 (7)	C9—C10	1.377 (8)
N2—C8	1.348 (8)	C9—H9A	0.9300
N3—C11	1.317 (10)	C10—C11	1.382 (12)
N3—C8	1.365 (6)	C10—H10A	0.9300
N4—C8	1.334 (8)	C11—H11A	0.9300
N4—H4B	0.8600	O1W—H1	0.8500
N4—H4C	0.8600	O1W—H2	0.8499
O1—C1	1.278 (5)	O2W—H3	0.8500
O2—C1	1.235 (6)	O2W—H4	0.8500
O3—C7	1.267 (6)	O3W—H5	0.8500
O4—C7	1.230 (6)	O3W—H6	0.8500
C1—C2	1.510 (6)		
			
N1—Ni1—N2	178.94 (18)	C2—C3—H3A	121.1
N1—Ni1—O1W	90.35 (15)	C4—C3—H3A	121.1
N2—Ni1—O1W	88.59 (17)	C5—C4—C3	120.4 (5)
N1—Ni1—O2W	93.46 (14)	C5—C4—H4A	119.8
N2—Ni1—O2W	87.59 (16)	C3—C4—H4A	119.8
O1W—Ni1—O2W	175.37 (12)	C6—C5—C4	118.5 (5)
N1—Ni1—O1	77.84 (13)	C6—C5—H5A	120.8
N2—Ni1—O1	102.19 (15)	C4—C5—H5A	120.8
O1W—Ni1—O1	88.48 (15)	N1—C6—C5	120.2 (4)
O2W—Ni1—O1	94.88 (13)	N1—C6—C7	112.9 (4)
N1—Ni1—O3	78.02 (13)	C5—C6—C7	126.9 (4)
N2—Ni1—O3	101.92 (15)	O4—C7—O3	124.2 (5)
O1W—Ni1—O3	90.07 (15)	O4—C7—C6	119.4 (5)
O2W—Ni1—O3	88.15 (13)	O3—C7—C6	116.4 (4)
O1—Ni1—O3	155.80 (13)	N4—C8—N2	119.3 (4)
C6—N1—C2	122.3 (4)	N4—C8—N3	117.0 (6)
C6—N1—Ni1	118.9 (3)	N2—C8—N3	123.7 (6)
C2—N1—Ni1	118.8 (3)	N2—C9—C10	123.4 (7)
C9—N2—C8	117.1 (5)	N2—C9—H9A	118.3
C9—N2—Ni1	115.7 (4)	C10—C9—H9A	118.3
C8—N2—Ni1	127.1 (4)	C9—C10—C11	115.1 (6)
C11—N3—C8	116.4 (6)	C9—C10—H10A	122.4
C8—N4—H4B	120.0	C11—C10—H10A	122.4
C8—N4—H4C	120.0	N3—C11—C10	124.2 (5)
H4B—N4—H4C	120.0	N3—C11—H11A	117.9
C1—O1—Ni1	114.9 (3)	C10—C11—H11A	117.9
C7—O3—Ni1	113.2 (3)	Ni1—O1W—H1	98.9
O2—C1—O1	125.5 (4)	Ni1—O1W—H2	121.3
O2—C1—C2	119.6 (4)	H1—O1W—H2	114.3
O1—C1—C2	114.8 (4)	Ni1—O2W—H3	109.9
N1—C2—C3	120.9 (4)	Ni1—O2W—H4	115.4
N1—C2—C1	113.2 (4)	H3—O2W—H4	116.5
C3—C2—C1	126.0 (4)	H5—O3W—H6	99.9
C2—C3—C4	117.7 (4)		
			
O1W—Ni1—N1—C6	92.7 (4)	O2—C1—C2—N1	175.1 (5)
O2W—Ni1—N1—C6	−84.7 (4)	O1—C1—C2—N1	−5.0 (6)
O1—Ni1—N1—C6	−179.0 (4)	O2—C1—C2—C3	−3.6 (8)
O3—Ni1—N1—C6	2.7 (3)	O1—C1—C2—C3	176.3 (5)
O1W—Ni1—N1—C2	−85.3 (3)	N1—C2—C3—C4	−0.4 (7)
O2W—Ni1—N1—C2	97.3 (3)	C1—C2—C3—C4	178.2 (5)
O1—Ni1—N1—C2	3.0 (3)	C2—C3—C4—C5	−0.5 (8)
O3—Ni1—N1—C2	−175.3 (4)	C3—C4—C5—C6	1.1 (9)
O1W—Ni1—N2—C9	−75.1 (4)	C2—N1—C6—C5	0.0 (7)
O2W—Ni1—N2—C9	102.3 (4)	Ni1—N1—C6—C5	−177.9 (4)
O1—Ni1—N2—C9	−163.2 (4)	C2—N1—C6—C7	179.2 (4)
O3—Ni1—N2—C9	14.7 (4)	Ni1—N1—C6—C7	1.3 (5)
O1W—Ni1—N2—C8	102.8 (5)	C4—C5—C6—N1	−0.8 (8)
O2W—Ni1—N2—C8	−79.8 (5)	C4—C5—C6—C7	−179.9 (5)
O1—Ni1—N2—C8	14.7 (5)	Ni1—O3—C7—O4	−169.6 (5)
O3—Ni1—N2—C8	−167.4 (4)	Ni1—O3—C7—C6	9.5 (5)
N1—Ni1—O1—C1	−5.9 (4)	N1—C6—C7—O4	171.6 (5)
N2—Ni1—O1—C1	173.0 (4)	C5—C6—C7—O4	−9.3 (8)
O1W—Ni1—O1—C1	84.8 (4)	N1—C6—C7—O3	−7.5 (6)
O2W—Ni1—O1—C1	−98.4 (4)	C5—C6—C7—O3	171.6 (5)
O3—Ni1—O1—C1	−2.0 (6)	C9—N2—C8—N4	−179.0 (5)
N1—Ni1—O3—C7	−6.9 (3)	Ni1—N2—C8—N4	3.2 (7)
N2—Ni1—O3—C7	174.2 (3)	C9—N2—C8—N3	2.3 (8)
O1W—Ni1—O3—C7	−97.2 (3)	Ni1—N2—C8—N3	−175.6 (4)
O2W—Ni1—O3—C7	87.1 (3)	C11—N3—C8—N4	179.1 (5)
O1—Ni1—O3—C7	−10.7 (6)	C11—N3—C8—N2	−2.1 (8)
Ni1—O1—C1—O2	−172.6 (5)	C8—N2—C9—C10	−1.2 (9)
Ni1—O1—C1—C2	7.4 (6)	Ni1—N2—C9—C10	176.9 (5)
C6—N1—C2—C3	0.6 (7)	N2—C9—C10—C11	0.0 (9)
Ni1—N1—C2—C3	178.6 (4)	C8—N3—C11—C10	0.8 (9)
C6—N1—C2—C1	−178.1 (4)	C9—C10—C11—N3	0.2 (9)
Ni1—N1—C2—C1	−0.2 (5)		

### Hydrogen-bond geometry (Å, °)

**Table:**

*D*—H···*A*	*D*—H	H···*A*	*D*···*A*	*D*—H···*A*
N4—H4B···O1	0.86	2.07	2.867 (5)	153
N4—H4C···O4^i^	0.86	2.12	2.973 (6)	173
O1W—H1···O4^ii^	0.85	2.29	2.906 (7)	130
O1W—H1···O3^ii^	0.85	2.37	3.152 (5)	152
O1W—H2···N3^iii^	0.85	2.03	2.835 (7)	158
O2W—H3···O2^iv^	0.85	1.93	2.777 (4)	178
O2W—H4···O3W^v^	0.85	1.84	2.685 (5)	173
O3W—H5···O2^vi^	0.85	1.89	2.736 (4)	177
O3W—H6···O4^ii^	0.85	2.15	2.964 (6)	159
O3W—H6···O3^ii^	0.85	2.56	3.253 (5)	140

Symmetry codes:  (i) *x*, *y*+1, *z*; (ii) −*x*+1, −*y*, −*z*; (iii) −*x*+1, −*y*+1, −*z*; (iv) *x*, −*y*+1/2, *z*−1/2; (v) *x*+1, *y*, *z*; (vi) −*x*+1, *y*−1/2, −*z*+1/2.

## References

[bb1] Aghabozorg, H., Bahrami, Z., Tabatabaie, M., Ghadermazi, M. & Attar Gharamaleki, J. (2007). *Acta Cryst.* E**63**, m2022–m2023.

[bb2] Aghabozorg, H., Manteghi, F. & Sheshmani, S. (2008). *J. Iran Chem. Soc.* **5**, 184–227.

[bb3] Altin, E., Kirchmaier, R. & Lentz, A. (2004). *Z. Kristallogr. New Cryst. Struct.* **219**, 35–36.

[bb4] Bruker (1998). *SADABS*, *SMART* and *SAINT-Plus* Bruker AXS Inc., Madison, Wisconsin, USA.

[bb5] Lee, J.-H. P., Lewis, B. D., Mendes, J. M., Turnbull, M. & Awwadi, F. (2003). *J. Coord. Chem.* **56**, 1425–1442.

[bb6] Li, Y.-G., Shi, D.-H., Zhu, H.-L., Yan, H. & Ng, S. W. (2007). *Inorg. Chim. Acta*, **360**, 2881–2889.

[bb7] Masaki, M. E., Prince, B. J. & Turnbull, M. M. (2002). *J. Coord. Chem.* **55**, 1337–1351.

[bb8] Ponticelli, G., Spanu, A., Cocco, M. T. & Onnis, V. (1999). *Transition Met. Chem.* **24**, 370–372.

[bb9] Prince, B. J., Turnbull, M. M. & Willett, R. D. (2003). *J. Coord. Chem.* **56**, 441–452.

[bb10] Sheldrick, G. M. (2008). *Acta Cryst.* A**64**, 112–122. 10.1107/S0108767307043930 18156677

[bb11] Tabatabaee, M., Aghabozorg, H., Attar Gharamaleki, J. & Sharif, M. A. (2009). *Acta Cryst.* E**65**, m473–m474. 10.1107/S1600536809011106 PMC2968778 21582403

[bb12] Tabatabaee, M., Hakimi, F., Roshani, M., Mirjalili, M. & Kavasi, H. R. (2008). *Acta Cryst.* E**64**, o2112. 10.1107/S1600536808032583 PMC2959642 21580976

[bb13] Tabatabaee, M., Masoodpour, L., Gassemzadeh, M. & Hakimi, F. (2009). *Acta Cryst.* E**65**, o2979. 10.1107/S1600536809045243 PMC2971878 21578719

[bb14] Tabatabaee, M., Sharif, M. A., Vakili, F. & Saheli, S. (2009). *J. Rare Earth*, **27**, 356–361.

